# Exploring the Leukemogenic Potential of GATA-1_S_, the Shorter Isoform of GATA-1: Novel Insights into Mechanisms Hampering Respiratory Chain Complex II Activity and Limiting Oxidative Phosphorylation Efficiency

**DOI:** 10.3390/antiox10101603

**Published:** 2021-10-12

**Authors:** Silvia Trombetti, Raffaele Sessa, Rosa Catapano, Laura Rinaldi, Alessandra Lo Bianco, Antonio Feliciello, Paola Izzo, Michela Grosso

**Affiliations:** Department of Molecular Medicine and Medical Biotechnology, University of Naples Federico II, Via S. Pansini 5, 80131 Naples, Italy; silvia.trombetti@unina.it (S.T.); raffaele.sessa@unina.it (R.S.); rosa.catapano3@studenti.unina.it (R.C.); laura.rinaldi2@unina.it (L.R.); alessandra.lobianco@unina.it (A.L.B.); antonio.feliciello@unina.it (A.F.); paola.izzo@unina.it (P.I.)

**Keywords:** myeloid leukemia, SDHC, respiratory chain complex II, GATA-1 isoforms, leukemogenesis, oxidative phosphorylation, oxidative stress, alternative splicing

## Abstract

GATA-1 is a key regulator of hematopoiesis. A balanced ratio of its two isoforms, GATA-1_FL_ and GATA-1_S_, contributes to normal hematopoiesis, whereas aberrant expression of GATA-1_S_ alters the differentiation/proliferation potential of hematopoietic precursors and represents a poor prognostic factor in myeloid leukemia. We previously reported that GATA-1_S_ over-expression correlates with high levels of the succinate dehydrogenase subunit C (SDHC). Alternative splicing variants of the SDHC transcript are over-expressed in several tumors and act as potent dominant negative inhibitors of SDH activity. With this in mind, we investigated the levels of SDHC variants and the oxidative mitochondrial metabolism in myeloid leukemia K562 cells over-expressing GATA-1 isoforms. Over-expression of SDHC variants accompanied by decreased SDH complex II activity and oxidative phosphorylation (OXPHOS) efficiency was found associated only with GATA-1_S_. Given the tumor suppressor role of SDH and the effects of OXPHOS limitations in leukemogenesis, identification of a link between GATA-1_S_ and impaired complex II activity unveils novel pro-leukemic mechanisms triggered by GATA-1_S_. Abnormal levels of GATA-1_S_ and SDHC variants were also found in an acute myeloid leukemia patient, thus supporting in vitro results. A better understanding of these mechanisms can contribute to identify novel promising therapeutic targets in myeloid leukemia.

## 1. Introduction

In recent decades, there has been increasing recognition of the role played by mitochondria in maintaining hematopoietic stem cell (HSC) functions, with deregulated mitochondrial activities often preceding malignant transformation of myeloid precursors [1,2]. Along with changes in mitochondrial metabolism, oxidative stress resulting from abnormal reactive oxygen species (ROS) production is a common signature of myeloid leukemic cells and contributes to development and clonal expansion of leukemia stem cells (LSCs) by upregulating pathways that sustain cell proliferation, survival, invasion, migration, and metabolic adaptation [1,3,4,5]. Furthermore, in comparison to normal cells, myeloid leukemia cells display an adaptive response to oxidative stress that is achieved by several mechanisms, including increasing antioxidant defenses representing a selective advantage that enhances cell survival under pro-oxidizing conditions [6,7,8,9,10,11].

Mitochondria are the main source of intracellular reactive oxygen species (ROS) that are mainly generated at complex I and at complex III of the respiratory chain (RC). The primary ROS produced in mitochondria is superoxide anion (O_2_^−^) that can be rapidly converted to hydrogen peroxide by superoxide dismutase (SOD) [12,13]. Notably, although previously largely neglected, more recent data points to a relevant role of complex II, the succinate dehydrogenase (SDH) or succinate-ubiquinone oxidoreductase (SQR) as a key redox regulator of ROS production [14,15,16,17]. Complex II plays a unique role since it represents a branching point of two essential mitochondrial pathways as being involved in both mitochondrial respiratory chain, where it reduces ubiquinone (CoQ) to ubiquinol (CoQH2), and in the Krebs cycle, where it oxidases succinate to fumarate, both processes being essential for the generation of ATP by OXPHOS [18]. This complex includes six protein subunits: SDHA, SDHB, SDHC, SDHD, SDHAF1, and SDHAF2, with the last two of them acting as associated accessory factors, whereas A and B subunits, containing the dehydrogenase catalytic domain, constitute the hydrophilic head that protrudes into the matrix compartment and C and D form the hydrophobic core embedded within the mitochondrial inner membrane. The catalytic core contains two types of prosthetic groups, FAD and Fe-S clusters, that participate to the electron transfer from succinate to CoQ whereas C and D subunits contain binding domains for a heme b560 moiety and two CoQ sites, the proximal high-affinity QP site and the distal low-affinity QD site. CoQ reduction occurs in two single-electron reactions, with the high-affinity QP site that markedly stabilizes the partially reduced semiquinone, thus allowing its complete reduction to CoQH2 [19,20]. Regarding the heme moiety and the QD site, their significance is still partly unclear. In fact, although the heme b560 is not required for CoQ reduction at the QP site, it supposedly takes part to the assembly of the entire complex in mammalian cells and mediate momentary electron transfer to the low-affinity QD site, thus serving as an electron pocket to avoid interactions between semiquinone CQ and O_2_ molecules, potentially leading to uncontrolled ROS generation [12,16,19].

Besides its well-established function in mitochondrial metabolism, more recently, starting from observations that SDH germline mutations have been found in several cancer types and contribute to abnormal intracellular and extracellular accumulation of succinate, many studies have highlighted a role of SDH as tumor suppressor and of its substrate succinate as oncometabolite [20,21]. An example is represented by hereditary pheochromocytoma, paraganglioma, renal cell carcinoma and gastrointestinal stromal tumors characterized by mutations in SDH subunits leading to the functional loss of complex II activity and, consequently, succinate accumulation, increased ROS generation, and decreased ATP production through OXPHOS. However, in this context, it is noteworthy that mutations occurring in different SDH genes lead to remarkable differences in clinical phenotype [22,23].

Loss of SDH activity leads to accumulation of succinate that acts as a competitive inhibitor of enzymes belonging to the class of α-ketoglutarate-dependent dioxygenase including histone demethylases and prolyl 4-hydroxylase (PHD). Notably, this latter enzyme is involved in post-translational regulation of hypoxia inducible factor 1α (HIF-1α) stability. Therefore, in this way, on the one hand, aberrant succinate levels can promote epigenetic alterations in cancer cells, on the other hand, following succinate inhibition of PHD-mediated degradation of HIF-1α, the HIF-1α survival pathway is constitutively activated leading to the transcription of genes that mediate the adaptive response to hypoxia, a common hallmark of cancer cells [14,24,25].

Defective complex II activity can also lead to a high rate of O_2_^−^ production, a condition that is linked to several disease scenarios associated with oxidative stress such as cancer and degenerative disorders. In this regard, several reports indicate that SDHC mutations result in increased O_2_^−^ production, oxidative stress and genomic instability, thus contributing to critical features of the malignant phenotype [26,27,28,29].

The human SDHC gene maps on the long arm of chromosome 1 and consists of six exons encoding for a 169-amino acid (aa) polypeptide corresponding to the full-length form of SDHC. Two main SDHC splicing variants have been described: the in-frame Δ3 ASV isoform lacking exon 3 with partial loss of the SDH oxidoreductase main activity region and the frameshift Δ5 ASV isoform, characterized by exon 5 skipping, loss of the heme binding domain and a 70-aa elongated C-terminal region. The Δ3 and Δ5 isoforms of SDHC have been associated with downregulation of SQR activity with respect to the full-length isoform, supporting the theory that ASV isoforms act as negative dominant variants of the full-length protein [30].

GATA-1 (Gene ID: 26239) is a master regulatory transcription factor of hematopoiesis. A balanced expression of its two isoforms, the full-length GATA-1_FL_ (NM_002049.3) and the shorter variant GATA-1_S_ (XM_024452363.1), is required in normal hematopoiesis whereas their dysregulation in favor of the short isoform alters the differentiation/proliferation potential of hematopoietic precursors and contributes to multi-step leukemogenesis [31,32,33,34,35,36,37]. However, despite much effort, there are several unanswered questions regarding the mechanistic principles linking GATA-1 dysregulation and hematological malignancies [36,38,39]. Starting from a recent study by our group showing that GATA-1 isoforms differently influences cell redox states, generation of mitochondrial O_2_^−^, expression levels of SDH subunits, and sensibility to apoptosis [6], we are now able to unveil a molecular mechanism through which unbalanced expression of GATA-1 isoforms could exert a leukemogenic role by impairing complex II activity and limiting OXPHOS efficiency.

## 2. Materials and Methods

### 2.1. Cell Culture

The human K562 erythroleukemia cell line obtained from European Collection of Authenticated Cell Cultures (EACC #89122407) was grown in RPMI 1640 medium supplemented with 10% fetal bovine serum (FBS) plus 4 mM glutamine, 10 U/mL penicillin, and 10 mg/mL streptomycin (purchased from Gibco, Thermo Fisher Scientific, Inc., Waltham, MA, USA) at 37 °C in a humidified 5% CO_2_^−^ containing atmosphere. Cells were cultured at 60–70% confluency for transient transfection experiments. Cells were routinely checked for mycoplasma contamination with the PCR Mycoplasma Test Kit (AppliChem, Darmstadt, Germany #A3744, 0020). Only cells negative for mycoplasma contamination were used.

### 2.2. Transient Transfection

Transient transfection experiments in K562 cells were performed as previously reported [6,40], using a mix containing 1 μg of p3XFLAG-GATA-1_FL_ (GATA-1_FL_ cells), p3XFLAG-GATA-1_S_ (GATA-1_S_ cells), or p3XFLAG-CMV empty vector (mock control) and 5 μL of lipofectamine 2000 as transfection reagent (Invitrogen, Carlsbad, CA, USA) in Opti-MEM medium (Invitrogen). Briefly, two hours before transfection, cells were seeded into six well plates at a density of 5 × 10^5^ in 2 mL of serum-free medium. Five hours after transfection, FBS was added to each well at final concentration of 10%. Forty-eight hours after transfection, cells were processed for total RNA and protein extraction.

For GATA-1_S_ knockdown experiments, K562 cells were seeded into a 24-well plate at a density of 2 × 10^5^ in in 100 μL of serum–free RPMI 1640 medium. A custom GATA-1_S_ small interfering RNA (GATA-1_S_ siRNA) was synthesized using the following target sequence: 5′-CCAGCCCAGTCTTTCAGGTG-3′ (Qiagen, GmbH, Hilden, Germany, #1027423) and transfected at final concentration of 50 and 100 nM [41]. The required amount of GATA-1_S_ and negative control siRNA (Qiagen, #1027310) was diluted in 100 μL of serum-free Opti-MEM (Invitrogen) and mixed with 6 μL of HiPerFect Transfection Reagent (Qiagen). The transfection mixtures were incubated for 15 min at room temperature and then added to the cells. Six hours after transfection, 400 μL of culture medium containing 10% FBS was added to each well. Forty-eight hours after siRNA transfection, K562 cells were collected for further analysis.

### 2.3. Protein Extraction

For total protein extraction, K562 cells were harvested and washed twice with 4 mL of cold 1× PBS by centrifugation at 3000× *g* for 10 min at 4 °C. Cells were lysated in 50 μL of lysis buffer (10% glycerol, 50 mM Tris-HCl pH 8.0, 150 mM NaCl, 0.1% NP-40, 1 mM EDTA pH 8, 0.5 μL of protein inhibitor cocktail mixture (Sigma-Aldrich, St. Louis, MI, USA) and incubated for 30 min on ice. Samples were collected after centrifugation at 10,000× *g* for 30 min at 4 °C. Protein concentration was evaluated spectrophotometrically, using the Bio-Rad protein assay reagent (Bio-Rad Laboratories, Hercules, CA, USA), according to the Bradford method [42].

### 2.4. Western Blot Analysis

Western blot analysis was performed on 15 μg of whole cell protein extracts as previously described [43]. Proteins were resolved on 10 or 14% SDS-page gels and transferred to nitrocellulose membranes by Trans-blot Turbo instrument (Bio-Rad). After blotting, filters were blocked and then hybridized at 4 °C for 1 hour and 30 min with the anti-FLAG antibody or overnight with the other primary antibodies. Primary antibodies were used at the following experimental conditions: FLAG (1:10,000 dilution; Sigma-Aldrich), GATA-1 (D24E4) (1:1000 dilution; Cell Signaling, Danvers, MA, USA #4589), SDHC (3E2) (1:500 dilution; Novus Biologicals Bio-Techne, Minneapolis, MN, USA #H00006391-M01), glutathione synthetase (1:20,000 dilution; Abcam, Cambridge, UK # ab124811), glutathione reductase (1:5000 dilution; Abcam, # ab124995). Membranes were washed thrice with 1x TBS-Tween 20 buffer for 5 min and incubated for 45 min with secondary antibodies conjugated to peroxidase (Bio-Rad Laboratories). The blots were visualized using the ECL Immobilon Western Chemiluminescent HRP-substrate system (Millipore, Darmstadt, Germany) and immunoreactive bands were detected by autoradiography according to the manufacturer’s instructions or by ChemiDoc XRS Image System (Bio-Rad Laboratories). Signals were subsequently normalized with antibodies anti-GAPDH (1:1000 dilution; Cell Signaling #2118) or anti α-actin (C-11) (1:10,000 dilution; Santa Cruz Biotechnology, Santa Cruz, CA, USA #sc-1615). Quantification of western blot bands was performed using the ImageJ software.

### 2.5. Total RNA Extraction

Total RNAs were extracted with the QIAzol reagent (Qiagen) from transfected K562 cells, as previously reported [44]. RNA quantization was performed spectrophotometrically, DNA contamination was excluded by gel electrophoresis on a 1.5% denaturing agarose gel in MOPS 1× buffer (20 mM MOPS pH 7.0, 8 mM Sodium Acetate, 1 mM EDTA pH 8.0.

### 2.6. Real-Time PCR Analysis

cDNA was synthesized from 500 ng of total RNA previously extracted from K562 cells or from bone marrow specimens using the QuantiTect Reverse Transcription Kit (Qiagen) and 2 μL of 7× gDNA Wipeout Buffer in a final volume of 14 μL. The reaction was incubated at 42 °C for 2 min and placed immediately on ice for complete removal of contaminating DNA. The reaction mixture was supplemented with 1 μL of RT primer mix, 4 μL of 5× Quantiscript RT Buffer and 1 μL of Quantiscript Reverse Transcriptase according to the kit protocol. This reaction was incubated at 42 °C for 3 min and at 95 °C for 3 min and subsequently used for real time RT-PCR procedures on a CFX96 Real-Time System (Bio-Rad Laboratories, Hercules, CA, USA). Primers for quantitative real time PCR analysis of *SDHC* transcripts were designed according to GenBank sequences: NG_012767.1 (SDHC), NM_003001.5 (full-length isoform), NM_001035512.2 (Δ3 ASV isoform), NM_001035511.2 (Δ5 ASV isoform). Primers used for HIF-1α were as previously reported [45]. GAPDH mRNA was used as endogenous control. All primer sequences are reported in Table 1. Each real-time PCR was performed in triplicate in a 20 μL reaction mix containing 10 μL of 2× SsoAdvanced Universal SYBR Green Supermix (Bio-Rad Laboratories), 0.38 μL of a 20 μM primer mix, 2 μL of cDNA (1/10 volume of RT-PCR product) and 7.62 μL of nuclease-free water. The cycling conditions were set up as follows: initial denaturation step at 98 °C for 30 s, followed by 40 cycles (95 °C for 15 s, 60 °C for 30 s) and a melting curve determined as previously reported [46]. The calibration curve was carried out for assessing the efficiency of the PCR reaction on at least three serial dilutions (1:10) of the reverse transcriptase products. Real-time PCR reactions were run in triplicates using the CFX96 Real-Time System (Bio-Rad Laboratories) and CT values were obtained from automated threshold analysis. Data were analyzed with the CFX Manager 3.0 software (Bio-Rad Laboratories GmbH, Munich, Germany) according to the manufacturer’s specifications.

In order to evaluate the Real-Time PCR reaction, a calibration curve was performed using three serial dilutions (1:10; 1:100; 1:1000) of RNA samples isolated from peripheral blood and then reverse-transcribed in cDNA. The acceptance range of the threshold cycles (Cq) obtained for the housekeeping GAPDH gene was set between 20.0 and 30.0. Therefore, samples with a GAPDH Cq > 30, indicative of poor quality of the starting RNA sample, were excluded from the analysis.

### 2.7. Measurement of the Enzymatic Activity of Complex II Succinate-Ubiquinone Oxidoreductase

Forty-eight hours after transient transfection, K562 cells were harvested and washed three times with 3 mL of cold 1× PBS by centrifugation at 3000× *g* for 10 min at 4 °C. Pellets were resuspended in 200 µL of Detergent/PBS solution 1X and incubated on ice for 30 min to allow proteins to dissolve. Samples were then centrifuged at 12,000× *g* for 20 min at 4 °C and the supernatant was collected and transferred into a clean Eppendorf tube. Evaluation of protein concentration was performed using the BCA Protein Quantification Kit (Abcam, #ab102536) according to the manufacturer’s instructions [43].

Complex II activity was measured using a Complex II Enzyme Activity Microplate Assay Kit (Abcam). Briefly, lysates (60 µg) were added to a mix (final volume 50 µL) containing incubation buffer to reach the concentration suggested by the manufacturer and hybridized on a 96-well microplate coated with an anti-Complex II monoclonal antibody in order to recognize and selectively capture the succinate dehydrogenase (SDH) complex; a positive control provided by the manufacturer was used to check hybridization efficiency.

Plates were incubated for 2 h at room temperature according to the manufacturer’s instructions. After removing the incubation solution, the wells were washed twice with 300 µL of Buffer 1×. Forty microliters of Lipid Mix were added to the wells and the mixtures were incubated for 30 min at room temperature.

Finally, 200 μL of substrate (activity solution) was added to each well and the optical density (λ = 600 nm) was measured at room temperature in a kinetic mode for 60 min on a Synergy H1 Hybrid Multi-Mode Microplate Reader (BioTek, Winooski, VT, USA). Data analysis was carried out according to the manufacturer’s instructions.

### 2.8. Seahorse Assay for Measurement of Cellular Respiration

Extracellular acidification rate (ECAR) and oxygen consumption rate (OCR) were measured in K562 cells transfected either with the empty vector (mock control), GATA-1_FL_ or GATA-1_S_ expression vectors using the Seahorse XF Cell Mito Stress test kit on a Seahorse XF24e flux analyzer (Agilent Technologies, Santa Clara, CA, USA) as previously described [47]. Forty-eight hours after transfection, 6 × 10^4^ cells were seeded in triplicate into poly-lysine-coated cell culture microplates (Agilent Technologies). The flux analysis protocol was as follows: ECAR and OCR were initially measured under basal conditions in XF media (non-buffered DMEM medium supplemented with 10 mM glucose, 2 mM  L-glutamine, and 1 mM sodium pyruvate) and after the sequential addition of oligomycin, a complex V inhibitor (1 µM), the mitochondrial uncoupler carbonyl-cyanide-p-trifluoromethoxyphenylhydrazone (FCCP) (1 µM) and lastly a combination of the complex I inhibitor rotenone (1 µM) and the complex III inhibitor antimycin A (1 µM). All measurements were normalized to the number of viable cells. Indices of mitochondrial respiratory function were calculated from the OCR profile: basal OCR (before addition of oligomycin), ATP-linked OCR (calculated as the difference between basal OCR rate and oligomycin-induced OCR rate), and maximal OCR (calculated as the difference of FCCP and rotenone + antimycin A rates). Spare respiratory capacity (SRC) was calculated as the difference between basal and maximal OCR. The results were analyzed in a Seahorse Report Generator (Agilent Technologies) [7,48].

### 2.9. Mitochondrial Mass Measurement

Mitochondrial mass was evaluated by cytofluorometry using Intracellular Fixation & Permeabilization Buffer Set (Thermo Fisher Scientific, Inc, # 88-8824-00.) according to a two-step protocol for intracellular proteins provided by the manufacturer. Forty-eight hours after transient transfection, K562 cells were harvested and washed twice with cold PBS by centrifugation at 3000 rpm for 10 min at room temperature. Cells were incubated for 30 min with Tom 20 antibody (1:200 dilution Cell Signaling, #42406) at room temperature and protected from light. Cells were then washed and incubated in PBS with a goat anti-rabbit IgG-FITC antibody (1:400 dilution, Alexa Fluor 488 #A11034) for 30 min at room temperature and protected from light. Stained cells were resuspended in an appropriate volume of Flow Cytometry Staining Buffer and the mean fluorescence intensity (MFI) was determined by flow cytometry using an Accuri C6 flow cytometer (BD Biosciences, San Jose, CA, USA) and BD Accuri C-flow software. Net fluorescence signals were evaluated after IgG (Sigma-Aldrich) background subtraction.

### 2.10. AML Patient Samples

Bone marrow aspiration specimens collected during routine diagnostic tests were obtained from a patient with AML. Informed consent for genetic studies was obtained in agreement with the Declaration of Helsinki. RNA extraction from bone marrow specimens was performed using the QIAzol (Qiagen) procedure [6].

### 2.11. Statistical Analysis

All data were assessed as the mean ± standard deviation (SD) of at least three separate experiments performed in triplicate. GraphPad Prism 7 (GraphPad Software, Inc., San Diego, CA, USA) was used for data analysis. Statistical differences were determinated through the one-way analysis of variance procedure followed by Dunnett’s multiple comparison test, comparing results between mock control and treated cells, Differences were statistically significant when *p* < 0.05 (*) (#) and highly significant when *p* < 0.0001 (**) (##) versus each respective mock control or untreated control group.

## 3. Results

### 3.1. Correlation between GATA-1 Isoforms and Expression Levels of SDHC Isoforms

To better elucidate the contribution of GATA-1 isoforms on SDHC expression, we firstly evaluated total SDHC expression levels on protein extracts from K562 cells transiently transfected either with p3xFlag expression vectors for specific GATA-1 isoforms (GATA-1_FL_ and GATA-1_S_) or an empty p3xFlag vector as negative control (Figure S1).

**Figure 1:**
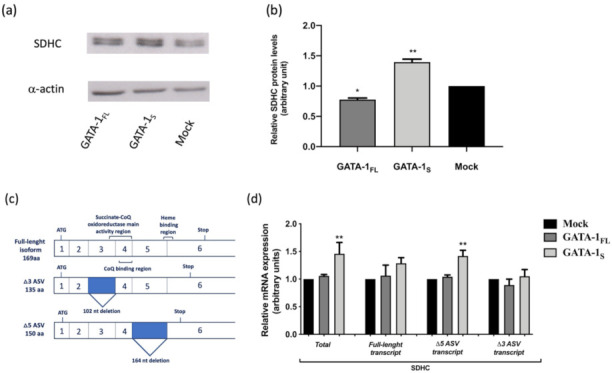
Western blot analysis for SDHC expression levels in total protein extracts obtained from K562 cells over-expressing GATA-1_FL_ and GATA-1_S_ isoforms and from a mock control. (**a**) Representative image of three independent experiments showing the presence of two SDHC-positive protein bands on the membrane possibly representing different SDHC isoforms. (**b**) Densitometric analysis of Western blot results showing total SDHC levels markedly increased only in K562 cells over-expressing the GATA-1_S_ isoform. For each sample, band intensities of the two SDHC signals, taken as a whole, were quantified from three independent experiments and normalized to α-actin used as a loading control. (**c**) Schematic representation of the alternative splicing mechanism generating SDHC variants (ASVs). Solid boxes and bars indicate the deleted exons and the corresponding protein domains, respectively. (**d**) Quantitative real-time PCR analysis of SDHC mRNA variants in cells over-expressing GATA-1 isoforms and in a mock control. mRNA expression levels were normalized against GAPDH. Results showed increased total SDHC transcript levels in cells over-expressing GATA-1_S_, thus confirming western blot analysis. Moreover, transcript-specific amplification revealed that SDHC abnormal expression in these cells was mostly due to the Δ5 ASV transcript. All data represent the mean ± SD of three independent experiments. Statistical analysis was performed by one-way ANOVA, followed by Dunnett’s multiple comparisons test, where appropriate. Differences were considered significant when *p* < 0.05 and highly significant when *p* < 0.0001. * *p* < 0.05, ** *p* < 0.0001 versus mock control.

Forty-eight hours after transfection, cells were harvested and SDHC protein levels were evaluated by western blot analysis on a 14% SDS-page gel (Figure 1).

As shown in Figure 1a,b, over-expression of the GATA-1_S_ isoform is accompanied by markedly increased SDHC protein level. On the contrary, cells over-expressing the GATA-1_FL_ isoform show reduced SDHC levels even with respect to the mock control. Notably, western blot analysis revealed the presence of two bands corresponding to the SDHC signal, suggestive of the presence of almost one of the two SDHC alternative splicing isoforms so far described, namely SDHC Δ3 ASV and SDHC Δ5 ASV, lacking succinate CoQ oxide reductase and heme b binding domains, respectively (Figure 1c).

**Figure 2:**
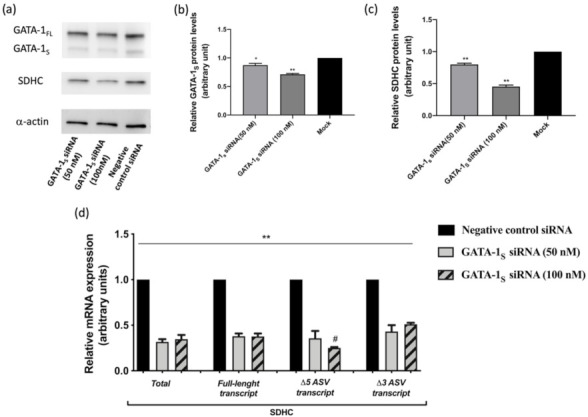
GATA-1_S_ knockdown experiments: (**a**) western blot analysis (10% SDS-page gel) of endogenous levels of GATA-1 isoforms and SDHC after K562 transfection with a custom GATA-1_S_ small interfering RNA (GATA-1_S_ siRNA) at final concentration of 50 and 100 nM. (**b**) Densitometric analysis of western blot results of GATA-1_S_ silenced protein. (**c**) Densitometric analysis of western blot results for SDHC after specific GATA-1_S_ siRNA transfection. (**d**) Quantitative real-time PCR analysis of SDHC mRNA variants in K562 cells previously transfected with two doses of specific GATA-1_S_ siRNA. mRNA expression levels were normalized against GAPDH and relative to negative control siRNA transfected cells. Results showed decreased total SDHC transcript levels in cells knocked down for GATA-1_S_, thus confirming western blot analysis. In addition, transcript-specific amplification revealed a more significant dose-dependent reduction for the Δ5 ASV isoform of SDHC following GATA-1_S_ silencing. All data represent the mean ± SD of three independent experiments. Statistical analysis was performed by one-way ANOVA, followed by Dunnett’s multiple comparisons test, where appropriate. Differences were considered significant when *p* < 0.05 and highly significant when *p* < 0.0001. * *p* < 0.05, ** *p* < 0.0001 versus negative control; # *p* < 0.05 versus lower dose of siRNA transfection.

This finding prompted us to evaluate the expression levels of the full-length and these two ASV SDHC isoforms in these cells. For this purpose, an isoform-specific quantitative real-time PCR assay was set-up to analyze different SDHC transcripts. Primers were designed to selectively amplify four amplicons in separate reactions corresponding to total SDHC transcripts and to the full-length, Δ3 and Δ5 transcripts, respectively. As shown in Figure 1d, RT-PCR analysis, besides confirming at the transcriptional level the western blot results obtained on total SDHC levels, allowed us to demonstrate that the increase in SDHC associated with GATA-1_S_ over-expression was mostly due to elevated transcript levels of its Δ5 ASV isoform. To further corroborate results from over-expression studies, knockdown of endogenous GATA-1_S_ levels was performed in K562 cells with two different doses of a custom-designed siRNA. Fifteen and thirty percent of GATA-1_S_ silencing was observed after 48 h post-transfection, at 50 and 100 nM GATA-1_S_ siRNA, respectively (Figure 2a).

No significant reduction was observed for the GATA-1_FL_ isoform, thus confirming that GATA-1_S_ siRNA specifically targets the shorter GATA-1 transcript. Western blot results showed a significant decrement of SDHC levels in a dose-dependent manner, reaching 50% reduction in cells transfected at the higher dose of GATA-1_S_ siRNA as compared to the siRNA negative control (Figure 2b,c). In addition, we also separately evaluated the expression levels of the full-length and the two ASV SDHC isoforms in these cells by quantitative RT-PCR assays. Interestingly, as shown in Figure 2d, RT-PCR results indicated a more significant dose-dependent reduction for the Δ5 ASV isoform of SDHC following GATA-1_S_ silencing. These findings are in full agreement with the over-expression studies indicating that GATA-1_S_ is specifically able to drive the abnormal expression of this SDHC Δ5 ASV isoform.

### 3.2. Effects of GATA-1 Isoforms on Mitochondrial Metabolism

#### 3.2.1. Measurement of SQR Activity

**Figure 3:**
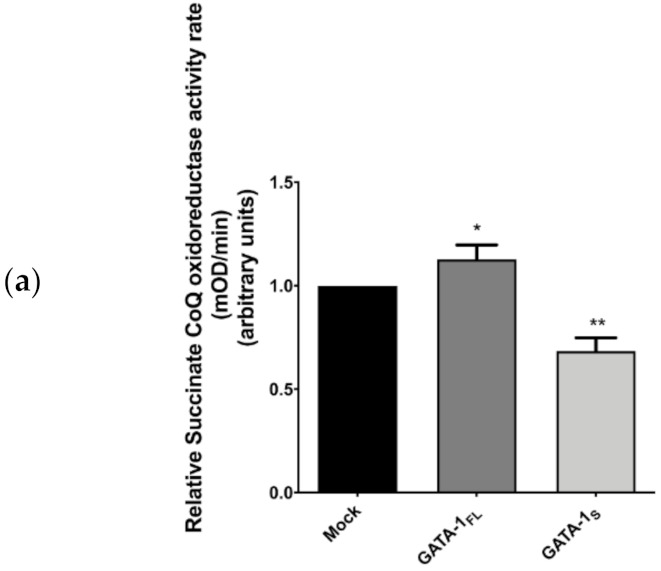

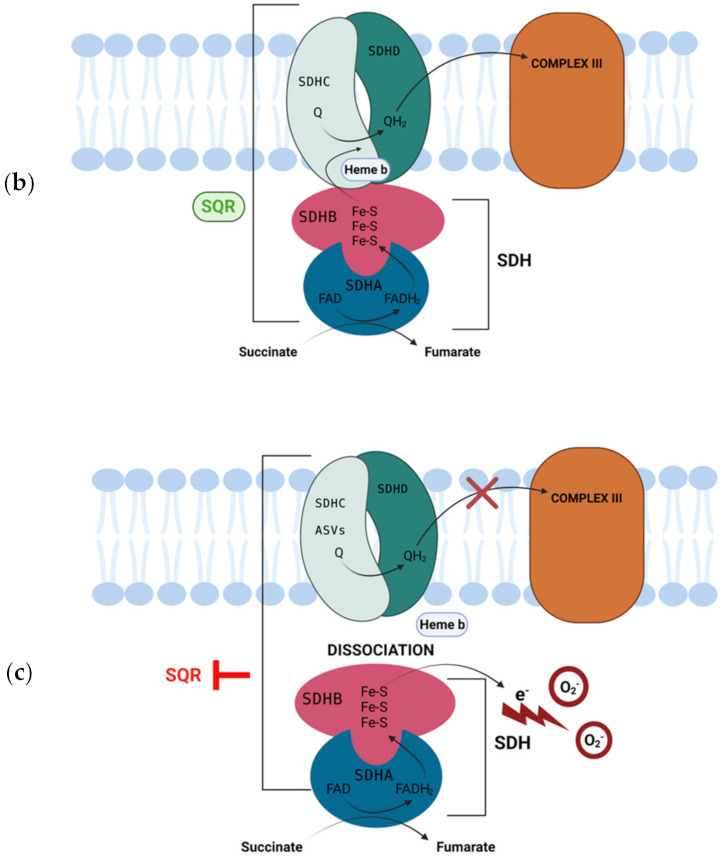
Enzymatic activity of succinate CoQ oxidoreductase (SQR) in K562 cells over expressing GATA-1 isoforms. (**a**) SQR activity detected on total cell lysates is expressed as OD absorbance/min/mg total protein. Data represent mean ± SD from three independent experiments. Differences were considered significant when * *p* < 0.05 and highly significant when ** *p* < 0.0001 versus mock control; (**b**,**c**) Schematic representation of complex II disassembly induced by over-expression of the SDHC Δ5 variant lacking the heme binding site with impaired SQR activity and increased O_2_^−^ production [8]. (Created with BioRender.com, accessed on 6 August 2021).

Based on these data and on the evidence that SDHC ASVs act as negative dominant of SDHC [28], we evaluated if the different expression profiles of SDHC variants detected in these cells could have an impact on the functional activity of complex II. To this aim, complex II (SQR) activity was assessed in K562 cells over-expressing either GATA-1_FL_ or GATA-1_S_. In agreement with literature data indicating reduced SQR activity in cells over-expressing the SDHC Δ5 ASV variant [30], our results showed that the activity of the succinate dehydrogenase complex significantly decreased to about 65% in cells over-expressing the GATA-1_S_ isoform compared to the mock control (Figure 3a). Conversely, a slight increase in SQR activity was detected associated with GATA-1_FL_ over-expression compared to the mock control (Figure 3a). As above reported, the SDHC Δ5 ASV variant lacks the heme b binding site. Although the significance of this heme moiety in complex II is still partly unclear and under debate, not being required for CoQ reduction [8,19], it is presumed to take part to the assembly of the complex in mammalian cells and contribute to reduce uncontrolled O_2_^−^ production. Therefore, it is expected that the reduced SQR activity observed in GATA-1_S_ cells over-expressing SDHC Δ5 may be related to impaired assembly of the SDH tetramer (Figure 3b,c) [7,11,13]. Importantly, this finding is in agreement and provides functional significance to our previous study demonstrating increased levels of mitochondrial O_2_^−^ in K562 cells over-expressing GATA-1_S_ [6].

#### 3.2.2. Evaluation of Cellular Energy Metabolism

**Figure 4:**
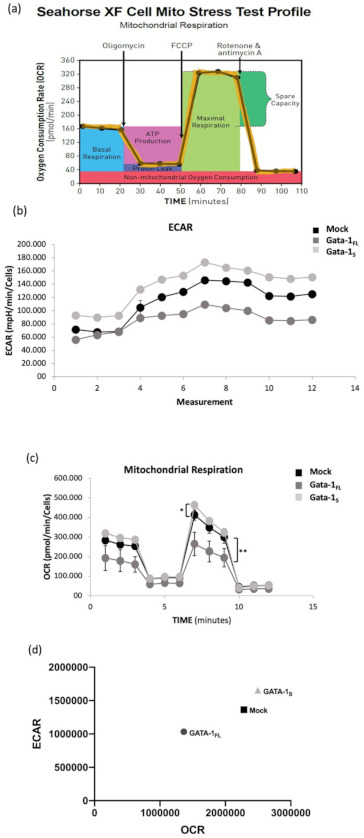

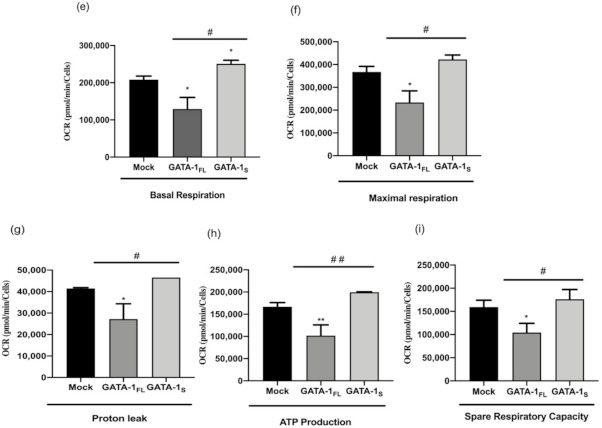
(**a**–**d**). Mitochondrial respiration rates measured by a Seahorse XFe assay in K562 cells overexpressing GATA-1 isoforms. (**a**) Schematic description of the experimental procedure; (**b**,**c**) variations in extracellular acidification (ECAR) and oxygen consumption rate (OCR, pmol/min/ng/mL) that were found increased in cells over-expressing GATA-1_S_ and decreased in response to GATA-1_FL_ over-expression as compared to the mock control. (**d**) Relationship between ECAR and OCR in intact cells under basal conditions. Data are presented as mean ± SEM. (**e**–**i**). Mitochondrial respiration rates measured by a Seahorse XFe assay in K562 cells overexpressing GATA-1 isoforms. Evaluation of basal (**e**) and maximal (**f**) respiration rates, proton leak (**g**), ATP production (**h**) and spare respiratory capacity (**i**) showing that GATA-1_FL_ over-expression is accompanied by reduced proton leak and ATP production. Conversely, over-expression of GATA-1_S_ is associated with higher respiration rate, enhanced proton leak, ATP production and spare respiratory capacity. These data are suggestive of GATA-1_S_ cells mainly being dependent on mitochondrial oxidative processes with respect to the full-length isoform. Data represent mean ± SD from three independent experiments. Differences were considered significant when *p* < 0.05 and highly significant when *p* < 0.0001. # *p* < 0.05, ## *p* < 0.0001 GATA-1_FL_ versus GATA-1_S_, * *p* < 0.05, ** *p* < 0.0001 versus mock control.

Given the role played by complex II in oxidative metabolism, we next asked whether the expression of GATA-1 isoforms could be related to differences in mitochondrial bioenergetics. To verify this hypothesis, we performed Mito Stress tests on a Seahorse flux analyzer that allows to measure real-time mitochondrial respiration of living cells (Figure 4a) thus providing information regarding energy metabolism in terms of extracellular acidification (ECAR), basal and maximal respiration, spare respiratory capacity (SRC), proton leak, and ATP production [49,50].

ECAR measurements, that are indirectly indicative of glycolytic flux [9], revealed higher ECAR levels and a rightward shift in ECAR relative to oxygen consumption rate (OCR) in GATA-1_S_ cells (Figure 4b–d), thus suggesting that these cells utilize glycolysis at higher rate than mitochondrial oxidative phosphorylation to meet energy requirements. In regard to mitochondrial respiration, results indicated that cells over-expressing GATA-1_S_ show an about two-fold increase in their basal respiration compared to GATA-1_FL_ cells and only a slighter increase compared to control; as far as other mitochondrial respiratory parameters are concerned, statistically lower SCR accompanied by a significant reduction in proton leak, maximal respiration and ATP production was observed in GATA-1_FL_ cells compared to both GATA-1_S_ and mock control (Figure 4e–h). Conversely, mitochondria in GATA-1_S_ cells showed enhanced basal and maximal respiration rates, but also higher proton leak, a sign of mitochondrial damage that underlines low coupling efficiency. Additionally, in apparent contrast with these data, enhanced ATP production was also detected in GATA-1_S_ cells, suggestive of a more efficient OXPHOS process (Figure 4h).

With the aim to clarify these conflicting findings, we assessed the mitochondrial mass in GATA-1_FL_ and GATA-1_S_ cells by detecting the levels of Tom20, a mitochondrial inner membrane marker protein [51].

**Figure 5:**
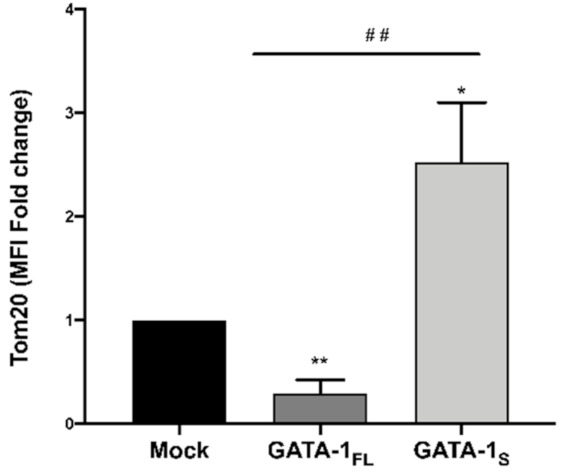
Total mitochondrial mass in cells over-expressing GATA-1 isoforms. Flow cytometry analysis was performed in fixed and permeabilized cells stained with Tom20 antibody 48 h after transfection. Results are indicative of increased mitochondrial network in cells over-expressing GATA-1_S_, thus confirming our previous findings (For more details see Riccio et al., ref [6]). Data represent mean ± SD from three independent experiments. Differences were considered significant when *p* < 0.05 and highly significant when *p* < 0.0001. ## *p* < 0.0001 GATA-1_FL_ versus GATA-1_S_, * *p* < 0.05, ** *p* < 0.0001 versus mock control.

**Figure 6:**
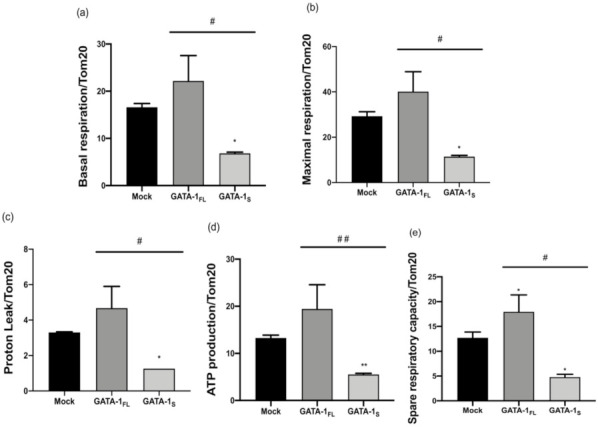
Cellular respiration rates related to mitochondrial mass in cells overexpressing GATA-1 isoforms. Basal (**a**) and maximal respiration (**b**) rates, proton leak (**c**), ATP production (**d**), and spare respiratory capacity (**e**) related to mitochondrial mass. Data represent mean ± SD from three independent experiments. Differences were considered significant when *p* < 0.05 and highly significant when *p* < 0.0001. # *p* < 0.05, ## *p* < 0.0001 GATA-1_FL_ versus GATA-1_S_, * *p* < 0.05, ** *p* < 0.0001 versus mock control.

The flow cytometry analysis was indicative of a dramatic increase of the mitochondrial mass in GATA-1_S_ cells oppositely to the reduced mass detected in GATA-1_FL_ cells (Figure 5). This finding was in agreement with our previous study that had also demonstrated an increased mitochondrial network in GATA-1_S_ cells strictly related to the increased size of these organelles, not to an increase in their number [6]. This finding is also supported by a large body of literature reporting increased mitochondrial mass in myeloid leukemia cells with respect to their normal counterpart [10,11,52]. Interestingly, when we normalized the respiration parameters to the size of the mitochondrial network, we found that, collectively, the indices of mitochondrial respiration in GATA-1_S_ cells were about 4-fold lower compared to either GATA-1_FL_ or mock control (Figure 6), thus suggesting that at least a part of the larger mitochondrial network in GATA-1_S_ cells is unable to contribute to the oxidative metabolism. As a whole, these data provided evidence that energy metabolism in cells over-expressing GATA-1_S_ is more dependent on glycolysis than on mitochondrial respiration due to reduced OXPHOS efficiency.

**Figure 7:**
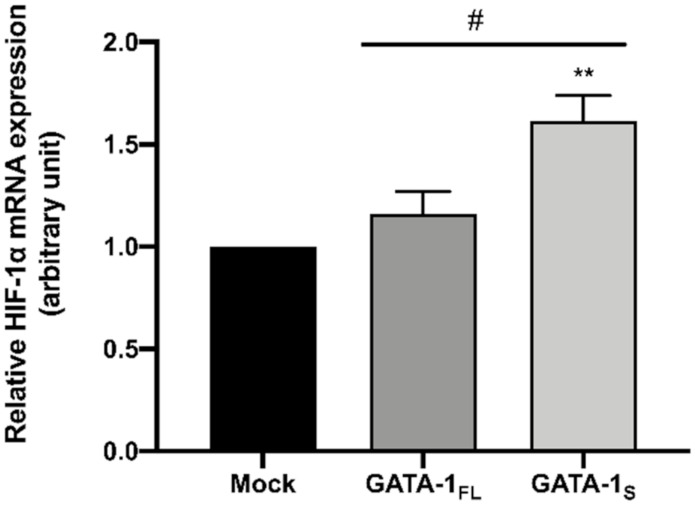
Quantitative real-time PCR analysis of HIF-1α transcript levels in cells over-expressing GATA-1 isoforms and in a mock control. mRNA expression levels were normalized against GAPDH. Results showed more significant increment of HIF-1α transcript levels in cells over-expressing GATA-1_S_, with respect to both GATA-1_FL_ and the mock control. Statistical analysis was performed by one-way ANOVA, followed by Dunnett’s multiple comparisons test, where appropriate. Differences were considered significant when *p* < 0.05 and highly significant when *p* < 0.0001. # *p* < 0.05, GATA-1_FL_ versus GATA-1_S_, ** *p* < 0.0001 versus mock control.

To further investigate on possible molecular mechanisms correlating GATA-1_S_ with enhanced glycolytic flux, we examined the expression levels of HIF-1α in cells over-expressing GATA-1 isoforms. As shown in Figure 7, high levels of HIF-1α transcript were observed only in cells over-expressing GATA-1_S_, thus suggesting HIF-1α gene as a transcriptional target of the shorter isoform of GATA-1.

### 3.3. Effects of GATA-1 Isoforms on Glutathione (GSH) Biosynthesis

**Figure 8:**
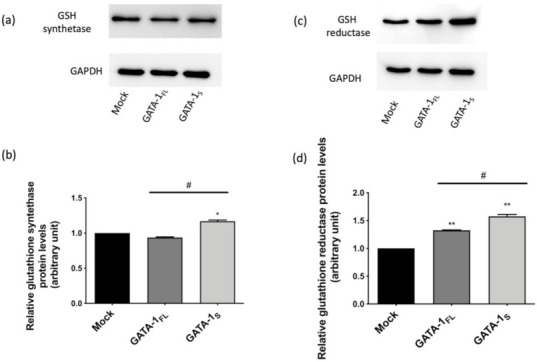
Expression levels of GSH biosynthetic enzymes. (**a**) Western blot analysis (10% SDS-page gels) of GSH synthetase expression levels in total protein extracts from a mock control and from cells over-expressing GATA-1_FL_ and GATA-1_S_, respectively; (**b**) densitometric analysis of western blot results. The figure shows representative results of three independent experiments; (**c**) Western blot analysis (10% SDS-page gels) of GSH reductase expression levels in total protein extracts from mock control and from cells over-expressing GATA-1_FL_ and GATA-1_S_, respectively; (**d**) densitometric analysis of western blot results. The figure shows representative results of three independent experiments. Differences were considered significant when *p* < 0.05 and highly significant when *p* < 0.0001. # *p* < 0.05, GATA-1_FL_ versus GATA-1_S_ * *p* < 0.05, ** *p* < 0.0001 versus mock control.

Previously, we had shown that GATA-1 isoforms are linked to different antioxidant defense abilities such as SOD and GSH levels that were found significantly increased in GATA-1_S_ cells. Moreover, a higher ratio of reduced GSH to its oxidized form (GSH/GSSG) was found in GATA-1_S_ cells compared to GATA-1_FL_ [6]. Besides acting as a ROS scavenger, GSH is involved in several processes including cellular proliferation, cell division and differentiation. Furthermore, excess GSH and dysregulated GSH metabolism are known to promote tumor progression, chemoresistance, and metastasis in a variety of malignancies [53,54]. Based on these observations, we asked whether GATA-1 isoforms differently contribute to the regulation of GSH metabolism. To this aim, we evaluated the levels of two enzymes involved in GSH biosynthesis, namely GSH synthetase (GSS) that catalyzes the second step of de novo GSH biosynthesis and has been found upregulated in several cancer types [54], and GSH reductase (GSR), a flavoprotein enzyme that regenerates GSH from GSSG [4]. In cells over-expressing GATA-1_S_ we found increased levels of GSH synthetase compared to both GATA-1_FL_ cells and the mock control (Figure 8a,b). Conversely, GSH reductase was increased in both cell types, even though at slightly higher level in the presence of over-expressed GATA-1_S_ (Figure 8c,d). These findings further reinforce the evidence of a stronger antioxidant capacity in GATA-1_S_ cells including enhanced expression of enzymes involved in antioxidant mechanisms as an adaptive response to oxidative stress in GATA-1_S_ cells that contribute to sustain cell survival under pro-oxidant conditions.

### 3.4. Expression Levels of SDHC ASV Isoforms in an AML Patient

**Figure 9:**
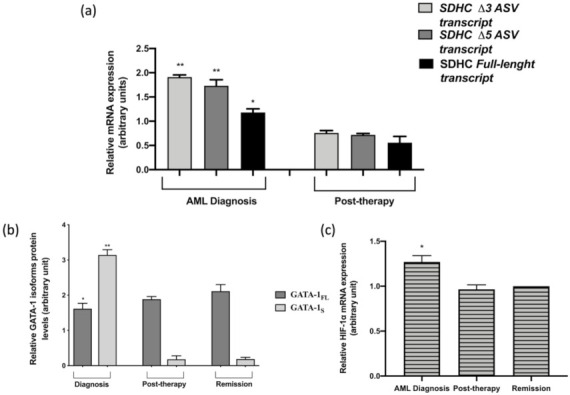
(**a**,**b**) Quantitative analysis of SDHC mRNA variant transcripts and GATA-1 isoforms protein levels from bone marrow specimens of an AML patient at diagnosis and post-therapy stages relative to the remission values. mRNA expression levels were normalized against GAPDH; (**c**) Quantitative real-time PCR analysis of HIF-1α transcript levels, normalized against GAPDH, in bone marrow samples obtained from the AML patient at diagnosis, post-therapy and remission stages. Results showed significant increment of HIF-1α transcript levels at the diagnosis stage with respect to post-therapy and remission stages. All data represent the mean ± SD of three independent experiments. Statistical analysis was performed by one-way ANOVA, followed by Dunnett’s multiple comparisons test, where appropriate. Differences were considered significant when *p* < 0.05 and highly significant when *p* < 0.0001. * *p* < 0.05, ** *p* < 0.0001 versus control.

Finally, to evaluate possible clinical implications to our findings, SDHC variants levels were measured at diagnosis (acute stage of the disease) and at remission in bone marrow specimens of an AML patient whom, in a previous study, we had analyzed for GATA-1 and SDHC expression levels [6]. In fact, in this patient at AML diagnosis (acute phase of the disease) we had shown dramatic elevated levels of both GATA-1_S_ and SDHC that were completely normalized at remission. Based on these previous observations [6], we were now able to add further insights into these features by demonstrating that the increased SDHC levels detected in this patient at diagnosis were mostly due to both SDHC Δ3 ASV and SDHC Δ5 ASV, with respect to the full-length isoform. Moreover, we herein report that reduction of SDHC levels in the post-therapy phase (remission stage) is mostly related to a dramatic reduction of these two ASV isoforms (Figure 9a). Notably, these findings are in full agreement with the results above reported for K562 cells, since even in the patient samples, over-expression of GATA-1_S_ (Figure 9b) correlates with increased expression levels of SDHC ASVs. As a whole, this study further supports the link between GATA-1_S_ and SDHC ASVs variants over-expression and its pro-leukemic significance.

Furthermore, our results in K562 cells showing enhanced glycolytic flux associated with GATA-1_S_ along with a large body of literature data reporting that increased glucose metabolism is a common feature of leukemia cells [55,56,57,58], linked to constitutively activation of HIF-1α pathway [3,59,60] prompted us to examine HIF-1α mRNA levels in these AML samples. Results showed significant increment of HIF-1α transcript levels at the diagnosis stage with respect to post-therapy and remission stages (Figure 9c). Interestingly, a similar trend had been observed for GATA-1_S_ protein levels in these same samples, thus further corroborating the relationship between GATA-1_S_ and HIF-1α levels detected in K562 cells. Notably, these findings also contribute to clarify the molecular mechanisms underlying the energy metabolic changes observed in cells over-expressing GATA-1_S_.

## 4. Discussion

Hematopoietic homeostasis is maintained by a refined equilibrium between self-renewal, proliferation, differentiation and survival of hematopoietic stem cells and their progeny with each process being supported by the balanced expression of several specific genes, regulated by different transcription factors. In this context, the GATA-1 factor plays a key role in regulating the expression of a large set of hematopoietic-related genes [32,61]. Therefore, not surprisingly, its dysregulated expression is emerging as a key factor in malignant hematopoiesis [32,62,63]. Two isoforms encoded from the same gene through alternative splicing or use of different translation start sites are known: the full-length GATA-1 isoform (GATA-1_FL_) and its shorter isoform (GATA-1_S_), lacking the N-terminal transactivation domain. In recent years these two variants have received extensive attention due to the evidence that they play opposite roles in the differentiation and proliferation processes, with the GATA-1_FL_ promoting the terminal differentiation and GATA-1_S_ mainly involved in the maintenance of the proliferative potency of hematopoietic precursors [6,31,64,65]. Therefore, it is evident that a balanced ratio between the two isoforms is required for normal hematopoiesis. On the contrary, unbalanced GATA-1_FL_/GATA-1_S_ expression with a prevalent expression of GATA-1_S_ has been found associated with several hematopoietic disorders including different acute and chronic myeloid leukemia subtypes where elevated GATA-1_S_ levels are recognized as a poor prognostic factor [62,66,67]. However, although several reports emphasize the pro-leukemic role of this isoform in hematological malignancies, mechanistic details still need clarification to decipher the role of GATA-1_S_ in malignant hematopoiesis.

Recently, we reported that GATA-1 isoforms differently contribute to the modulation of redox microenvironment and apoptosis sensitivity in myeloid cells [6]. Variations both in ROS levels and in intracellular compartmentation were found associated with the expression of specific GATA-1 isoforms: cytosolic ROS had resulted to be dramatically increased only in GATA-1_FL_ cells alongside higher mitochondrial superoxide concentrations. We also found that the lower oxidative status in GATA-1_S_ cells was related to enhanced antioxidant defenses, including increased SOD1 and GSH content. Furthermore, we also demonstrated that GATA-1s over-expression was accompanied by markedly increased levels of SDHC, a subunit of the respiratory chain SQR complex, without any appreciable change in the other three subunits (SDHA, SDHB, SDHD) of the SQR tetramer [6]. In this regard, it is to be noted that, although it has long been assumed that mitochondrial ROS can only be produced at complexes I and III, more recent data pointed out that complex II has a role in ROS production, thus significantly contributing to the mitochondrial control of apoptosis and cell proliferation [12,13,15,16,17]. In this context, a growing body of research has focused on the role of SDH as a tumor suppressor factor and the relationship between complex II dysregulation and tumorigenesis consequent to chronic ROS elevation and impaired regulation of apoptosis [18,20,21]. Accordingly, germline mutations in SDH genes resulting in impaired SDH activity have been found in several tumor types including pheochromocytoma, paraganglioma, gastrointestinal carcinoma, renal cell carcinoma, thyroid carcinoma, neuroblastoma, and breast cancer along with altered SDH epigenetic and post-translational mechanisms of regulation [22,23,27,28,29]. Furthermore, the oncogenic activity of these mutations has been associated with a high production of O_2_^−^ that may be responsible for the genomic instability of cancer cells. Therefore, in light of all these findings, we speculated on a role of GATA-1_S_ in the regulation of complex II activity and its potential pro-leukemic significance.

Two isoforms of SDHC are produced by alternative splicing mechanisms: Δ3 alternative splicing variant (Δ3 ASV), which lacks exon 3 encoding the main region of oxidoreductase activity of complex II, and Δ5 ASV, defective of the exon 5 encoding the binding region for the heme-b 560 group. Δ3 and Δ5 ASVs are both ubiquitously expressed even at almost two-fold lower level than the full-length mRNA [26,30]. Conversely, these isoforms are over-expressed in different tumor lines, such as HCT-15 colorectal adenocarcinoma cells, where the increase in the expression of SDHC Δ3 and Δ5 ASVs was found associated with reduced SDH activity and increased production of O_2_^−^ [23,27]. Therefore, based on these observations, we determined the expression levels of these two SDHC ASVs in K562 over-expressing GATA-1 isoforms and found that upregulation of SDHC in GATA-1_S_ cells was accompanied by a prevalence of the SDHC Δ5 ASV transcript compared to GATA-1_FL_ cells. In addition, expression levels of SDHC Δ5 in GATA-1_S_ cells inversely correlated with the SQR activity of complex II. Interestingly, these results are in agreement with the dominant-negative inhibition so far reported for the SDHC Δ5 isoform on its full-length variant [30] as well as with our previous findings showing enhanced production of O_2_^−^ associated with GATA-1_S_ over-expression [6]. Conversely, GATA-1_S_ knocking down was accompanied by reduced expression of SDHC, with particular regard to its Δ5 ASV variant. Importantly, as a whole, these findings strongly corroborate our hypothesis that the leukemogenic potential of GATA-1_S_ can be related to complex II dysfunction.

In line with these results, we next asked whether the expression of GATA-1 isoforms could differently influence the contribution of mitochondria to cellular metabolism. Almost unexpectedly, assessment of cellular respiration rates normalized to mitochondrial content revealed a more efficient mitochondrial metabolic activity in GATA-1_FL_ cells. On the contrary, despite the larger mitochondrial content, GATA-1_S_ cells showed higher ECAR values along with reduced rates of mitochondrial respiration and ATP production. As a whole, these metabolic parameters suggest that, at least in part, the larger mitochondrial network in GATA-1_S_ cells is hindered to efficiently contribute to the oxidative metabolism due to molecular mechanisms limiting OXPHOS. In this regard, it is to be noted that these findings are in agreement with a large body of evidence indicating that, compared with their normal counterpart, AML cells display a larger mitochondrial network without increased respiratory chain activity alongside a lower spare reserve capacity that makes them more susceptible to oxidative stress [1,2,3,7,9,50]. In addition, the higher ECAR values detected in GATA-1_S_ cells correlates with an enhanced glycolytic flux that invokes the pseudo-hypoxic phenotype occurring when the HIF-1α pathway is constitutively activated, regardless of oxygen levels, a condition that, as above mentioned, characterizes cancer cells and can be driven by loss of complex II activity [24,68]. Based on these observations, we were thus prompted to evaluate HIF-1α levels in these cells and found high levels of HIF-1α only in cells over-expressing GATA-1_S_. Although more studies are required to better clarify the regulatory network involving GATA-1 and HIF-1α in normal and aberrant hematopoiesis, these finding shed new light on the molecular mechanisms leading to metabolic rewiring in leukemia cells.

Literature data also indicate that over-expression of defective SDHC variants is associated with increased production of O_2_^−^, enhanced oxidative stress and reinforced antioxidant defense systems that contribute to limit oxidative damage caused by excessive ROS production [26,27,28]. Interestingly, these findings actually resemble what we had previously seen in cells over-expressing GATA-1_S_ showing elevated mitochondrial O_2_^−^ levels along with enhanced antioxidant defenses due to increased levels of GSH and SOD1 [6]. In the present study we add further light on the leukemogenic potential of GATA-1_S_ by providing evidence that the altered redox state in these cells can be associated with defective SDHC expression, impaired complex II activity, and OXPHOS efficiency. Herein, we also demonstrate enhanced expression of enzymes involved in GSH biosynthesis that contribute to reinforce the antioxidant capacity in GATA-1_S_ cells thus providing further insights into the mechanisms triggered in these cells to escape excessive ROS production.

Moreover, another aspect that needs to be considered is the relevant contribution of dysregulated alternative splicing mechanisms underlying both GATA-1_S_ and SDHC ASVs expression in promoting leukemia development. In this context it is interesting to note that, whereas alternative splicing variants extensively contributes to the regulation of organ development and cell differentiation programs, disruption of the splicing machinery is associated with human diseases and can contribute to oncogenesis and development of drug resistance in cancer cells [69,70]. Furthermore, as largely reviewed, maintenance of normal isoform ratios is crucial to control lineage commitment and progenitor maturation in normal hematopoiesis [71,72,73]. Expectedly, based on these observations, a more comprehensive understanding of the mechanisms and the factors regulating SDHC alternative splicing could help identify novel therapeutic targets aimed at limiting the proliferative and oncogenic potential in leukemic cells. Although it is currently unknown if GATA-1_S_ has a direct role in the alteration of the splicing machinery, our results clearly indicate that, along with unbalanced GATA-1 isoform ratio, aberrant expression of SDHC ASVs emerges as a leukemia-promoting factor. Taken as a whole, our results shed further light on the different roles played by GATA-1 isoforms in metabolic rewiring of mitochondria that could contribute to modulate the cellular redox environment to sustain either differentiation or proliferation programs in normal or malignant hematopoiesis.

## 5. Conclusions

The mitochondrial network acts as a central hub that directly or indirectly controls many cellular processes including proliferation, ATP synthesis, and cell death through the complex integration of metabolic, bioenergetics, and redox signals. Metabolic reprogramming of these organelles contributes to leukemia development and progression. In this study we describe a link between the expression levels of GATA-1 isoforms and SDHC ASVs levels, leading to the regulation of respiratory chain complex II activity and reduced oxidative phosphorylation efficiency, thus paving the way for the understanding of the molecular mechanisms by which GATA-1_S_ could contribute to leukemia onset and development. Improved understanding of the factors regulating alternative splicing could help to decipher the role of GATA-1_S_ in myeloid leukemia and to identify novel mitochondrial vulnerabilities in LSCs as promising therapeutic targets for this disease.

## Figures and Tables

**Table 1:** Primer sequences used for quantitative Real-time PCR analysis.

Transcript	Accession Number	Primer	Sequence 5′-3′	Amplicon Size
SDHC	NG_012767.1	For 1	CACTTCCGTCCAGACCGGA	100 bp
		Rev 1	CTGATACAGAGCTGAGGGCTAA	
SDHC full-length	NM_003001.5	For 2	TCTGTATCAGAAATGCTGTTCC	183 bp
		Rev 2	GAGACCCCTGCACTCAAAGC	
SDHC Δ 3 ASV	NM_001035512.2 (AB211234.1)	For 3	GCTCTGTATCAGAAATTGGTCT	250 bp
		Rev 3	GTCCCACATCAAGTGTCGGA	
SDHC Δ 5 ASV	NM_001035511.2 (AB211235.1)	For 2	TCTGTATCAGAAATGCTGTTCC	187 bp
		Rev 4	GGTCCCACATCTGCACTCAA	
HIF-1α	NM_001530.4	For	TCCAAGAAGCCCTAACGTGT	179 bp
		Rev	TGATCGTCTGGCTGCTGTAA	
GAPDH	NM_002046.7	For	GAGCCACATCGCTCAGACAC	116 bp
		Rev	GGCAACAATATCCACTTTACCA	

## Data Availability

Data is contained within the article.

## Supplementary Materials

The following are available online at https://www.mdpi.com/article/10.3390/antiox10101603/s1, Figure S1: Western blot analysis of the expression levels of GATA-1 isoforms in total protein lysates from K562 cells after transient transfection with either FLAG-tagged GATA-1FL (48 kD), GATA-1S (38 kD) isoforms or empty vector (mock control).
