# Supplementary material for: Exploring the Leukemogenic Potential of GATA-1S, the Shorter Isoform of GATA-1: Novel Insights into Mechanisms Hampering Respiratory Chain Complex II Activity and Limiting Oxidative Phosphorylation Efficiency
Source: Antioxidants (Basel). 2021 Oct 12;10(10):1603. doi: 10.3390/antiox10101603 (PMC8533167; doi:10.3390/antiox10101603)
Supplement: Supplementary file 1 [file antioxidants-10-01603-s001.zip › antioxidants-1387552-supplementary.pdf]

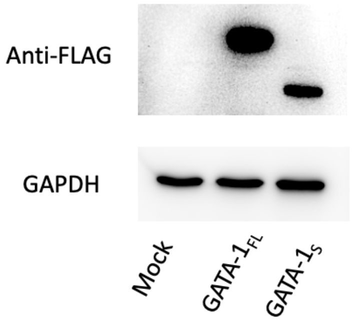

Figure S1. Western blot analysis of the expression levels of GATA‐1 isoforms in total protein lysates from K562 cells after transient transfection with either FLAG‐tagged GATA-1FL (48 kD), GATA-1S (38 kD) isoforms or empty vector (mock control).
